# Inhibition of LRRK2 Attenuates Depression-Related Symptoms in Mice with Moderate Traumatic Brain Injury

**DOI:** 10.3390/cells12071040

**Published:** 2023-03-29

**Authors:** Alessia Filippone, Laura Cucinotta, Valentina Bova, Marika Lanza, Giovanna Casili, Irene Paterniti, Michela Campolo, Salvatore Cuzzocrea, Emanuela Esposito

**Affiliations:** Department of Chemical, Biological, Pharmaceutical and Environmental Sciences, Viale Stagno d’Alcontres, 31, 98166 Messina, Italy; afilippone@unime.it (A.F.); laura.cucinotta@unime.it (L.C.); valentina.bova@unime.it (V.B.); mlanza@unime.it (M.L.); gcasili@unime.it (G.C.); ipaterniti@unime.it (I.P.); campolom@unime.it (M.C.); salvator@unime.it (S.C.)

**Keywords:** depression, moderate traumatic brain injury, neuroinflammation

## Abstract

Moderate traumatic brain injury (mTBI) has been associated with emotional dysregulation such as loss of consciousness, post-traumatic amnesia and major depressive disorder. The gene Leucine-rich repeat kinase 2 (LRRK2) is involved in protein synthesis and degradation, apoptosis, inflammation and oxidative stress, processes that trigger mTBI. The aim of this study was to investigate the role of LRRK2 in reducing depression-related symptoms after mTBI and to determine whether inhibition of LRRK2 mediated by PF-06447475 could have antidepressant effects. Moderate traumatic brain injury was induced by controlled cortical impact (CCI) and mice were treated with PF-06447475 at doses of 1, 2.5 and 5 mg/kg once daily for 14 days. We performed histological, immunohistochemical and molecular analyses of brain tissue 24 days after mTBI. Furthermore, the tissue changes found in the hippocampus and amygdala confirmed the depression-like behavior. PF-treatment with 06447475 significantly reduced the histological damage and behavioral disturbances. Thus, this study has shown that mTBI induction promotes the development of depression-like behavioral changes. LRRK2 inhibition showed an antidepressant effect and restored the changes in the copper/glutamate/N-methyl-D-aspartic acid receptor (Cu/NMDAR) system.

## 1. Introduction

Moderate traumatic brain injury (mTBI) occurs after various accidental events such as collisions, car accidents, and others. The World Health Organization Collaborating Centre (WHO) Working Group on Moderate Traumatic Brain Injury has given about 30 different definitions used in the conventional literature [1], with the well-known definition referring to mTBI as “an acute brain injury resulting from mechanical injury to the head by external physical forces” [2]. Although external forces cause direct traumatic injury at the site of the lesion, which initiates pathophysiological processes, many psychological and neurological sequelae occur. mTBI has been associated with emotional dysregulation such as loss of consciousness, post-traumatic amnesia and major depressive disorder (MDD) in over 40% of interested individuals [3], and incidence levels reflect established relationships between mTBI and physical injury to the brain [4]. MDD result in significant neurological changes in several brain regions such as the prefrontal cortex (PFC), amygdala and hippocampus (HP) that regulate cognitive functions such as learning and memory [5]. 

Here, initial neuroinflammation triggered by the production and release of inflammatory cytokines such as interleukins (ILs) and chemokines by glial cells influences plastic restructuring in the hippocampus and cortical circuitry, affecting tissue architecture and copper homeostasis (Cu). Copper is an essential microelement for brain development and functionality [6]. Recently, several clinical studies have shown that elevated concentrations of free Cu in serum are associated with cognitive deficits, neurodegenerative diseases such as Alzheimer’s disease (AD) [7] and changes in the glutamate/N-methyl-D-aspartic acid receptor (NMDAR) in HP individuals diagnosed with MDD [8]. Although canonical antidepressants such as esketamine (or Spravato^®^) and monoamine transporter inhibitors are widely used [9], there is as yet no promising clinical approach to prevent the long-term consequences of mTBI.

Leucine-rich repeat kinase 2 (LRRK2), the known mutant gene involved in both familial and sporadic Parkinson’s disease (PD), is a multidomain protein (2527 amino acids, 286 kDa) widely expressed in circulating immune cells, the liver, the kidney and the brain [10]. In the brain, LRRK2 is expressed in several regions, including the cortex, hippocampus, striatum and substantia nigra pars compacta. LRRK2 activity increases following phosphorylation at various sites (including serine 935) and is able to promote post-ischemic apoptotic cell death by modulating tau phosphorylation during experimental cerebral ischemia. In addition, LRRK2 affects protein synthesis and degradation, apoptosis, inflammation and oxidative stress, processes that trigger mTBI. It has been reported that inhibition of LRRK2 with the antagonist PF-475 alleviated neuronal apoptosis, brain oedema and neurological deficits induced by traumatic injury [11,12]. Moreover, pharmacological inhibitors of LRRK2 not only modulate neuronal cell death and neuroinflammation, but also prevent the behavioral defects in mice induced by controlled cortical impact injury (CCI) [13]. In addition, LRRK2 was found to be increased in neurons and microglia following weight loss after brain injury, but the exact mechanism and physiological significance of this phenomenon remained unclear. These observations suggest that upregulation of LRRK2 contributes to secondary brain injury following TBI, although little is known about the exact molecular mechanisms involved in LRRK2-mediated neurotoxicity [14].

Therefore, in this study, we aimed to delineate the role of LRRK2 in reducing depression-related symptoms in a mouse model of mTBI and to determine whether Cu metabolism, glial cells and glutamate circuitry are involved in the underlying mechanisms by which the LRRK2 antagonist PF-475 exerts its antidepressant effect.

## 2. Materials and Methods

### 2.1. Materials

PF-06447475 (also PF-475) was purchased from MedChemExpress LLC (Monmouth Junction, NJ, USA; # HY-12477). All solutions were dissolved in non-pyrogenic saline (0.9% NaCl; Baxter, Liverpool, UK). Sigma-Aldrich (Milan, Italy) supplied all compounds, unless otherwise stated. 

### 2.2. Animals

CD1 mice (male, 10–12 weeks of age, 25–30 g, Envigo, Italy) were housed in stainless cages and maintained under a 12:12 h light/dark cycle, T 21 ± 1 °C, ± 5% humidity. Standard laboratory diet and tap water were available ad libitum. Animal care was in compliance with Italian regulations on the use of animals (D.M.116,192) and Directive legislation (EU) (2010/63/EU) amended by Regulation (EU) 2019/1010. The animal protocol was declared exempt, by an institutional review board University of Messina Review Board for the care of animals, in compliance with Italian regulations on the protection of animals (n° 399/2019-PR released on 2019).

### 2.3. Controlled Cortical Impact (CCI) Experimental TBI

Mice were subjected to a moderate traumatic brain injury by a controlled cortical impact by using the controlled impactor device Impact OneTM Stereotaxic impactor for CCI (Leica, Milan, Italy), as previously described [15]. A craniotomy of the right hemisphere was executed using a micro-motor handpiece and drill (UGO Basile SRL, Comerio Varese, Italy), among the sagittal suture and the coronal ridge. Once the resultant bone flap was removed, a cortical contusion (tip diameter: 4 mm; cortical contusion depth: 3 mm; impact velocity: 1.5 m/s) was made on the exposed cortex using the controlled impactor device. Immediately after injury, the skin incision was secured with nylon sutures.

### 2.4. Experimental Design

Mice were divided following simple randomization and partial blinding methods, as previously described [16]. To establish the minimum number of mice for every technique, the statistical test “ANOVA: Fixed effect, omnibus one-way” was carried out with G-power software. This test raised a sample size equal to n = 10 mice for each technique.

- Non-moderate TBI: mice were subjected to identical surgical procedures except for CCI and were kept under anesthesia for the duration of the experiment;
- Moderate TBI group: mice were subjected to CCI (n = 10) and were kept under anesthesia for the duration of the experiment;
- Moderate TBI + PF-475 (dose of 1 mg/kg) group: mice were subjected to CCI and subsequently treated with PF-475, i.p. administration for 14 days, 10 days after mTBI;
- Moderate TBI + PF-475 (dose of 2.5 mg/kg) group: mice were subjected to CCI and subsequently treated with PF-475, i.p. administration for 14 days, 10 days after mTBI;
- Moderate TBI + PF-475 (dose of 5 mg/kg) group: mice were subjected to CCI and subsequently treated with PF-475, i.p. administration for 14 days, 10 days after mTBI.

**Scheme 1:**
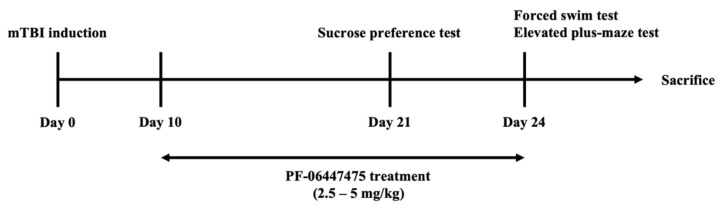
Experimental timeline.

To investigate the antidepressant-like effects of PF-475 following mTBI in mice, animals received PF-475 once daily for 14 consecutive days. Behavioral tests such as sucrose preference test, forced swim test and elevated plus-maze test were used to assess depression-like behaviors in mice. After 24 days from mTBI, brain areas were divided, collected and used for analyses (Scheme 1). In our laboratory, a preliminary dose-response study was performed to obtain the experimental doses to be used. Based on previous in vivo studies, we chose PF-475 as the route of administration [12,14]. PF-475 was prepared in dimethyl sulfoxide (DMSO) and diluted with 0.9% saline to obtain a final DMSO concentration of 1%.

### 2.5. Behavioral Tests

For acclimation, mice were placed in behavior rooms for 5 min a day for 2 days prior to the onset of behavioral tests. The behavioral tests were conducted by three different reliable expert observers blinded to the injury status of the animals, as described below.

#### 2.5.1. Sucrose Preference Test (SPT)

The sucrose preference test was performed 21 days after mTBI, as previously described [17]. Animals were acclimated with two identical water bottles in the cages and then were trained to consume 2% sucrose solution for 3 days to reduce the reaction to novelty and establish baseline sucrose preference. Three days later, after 18 h food and water deprivation (animals only received either drugs or vehicle during the food and water deprivation), each animal was given two bottles (2% sucrose solution and fresh tap water) for 1 h. Immediately before and after the test, we recorded the amount of the sucrose solution or water consumed by weighing the bottles. The percentage of sucrose preference was used as an indicator of anhedonia behavior.

#### 2.5.2. Forced Swim Test (FST)

The forced swim test was performed 24 days after mTBI, as previously described [17]. In brief, the experiment was executed in transparent glass cylinders (25 cm height, 10 cm diameter) full of fresh tap water (25 ± 1 °C; 15 cm deep). We measured the time of immobility during the last 4 min of the 6-min testing period, when the animals motionlessly floated on the water without struggling. At the end of test, mice were dried with towels and placed gently near an electric heater for 20 min. The immobility was used as an indicator of behavior despair.

#### 2.5.3. Elevated Plus-Maze (EPM) Test

The elevated plus-maze was performed 24 days after mTBI, as previously described [18]. The EPM apparatus was made of metal and black plastic and consisted of two opposite open arms (45 cm × 10 cm) without side walls and two enclosed arms (45 cm × 10 cm × 30 cm) with sides and end walls, extending from a central square (10 cm × 10 cm). The maze was elevated to a height of 60 cm above the floor and placed in a dimly lit room (8 lux as measured at the center of the maze). At the onset of the test, the animals were placed in the center of the EPM, facing towards an open arm, and the experimenter recorded the number of open and closed arm entries, as well as the time spent on either type of the arms, during a 10 min test period. An entry was counted when animal was on an arm with all four paws. All experiments were recorded on video as well, and later analyzed by each minute. The percentage of entries onto open arms from total arm entries and the percentage of time spent on the open arms were taken as measures of anxiety. In addition, the total number of entries was scored as a measure of locomotor activity.

### 2.6. Histological Evaluation

Brain tissue was removed and divided into two sagittal sections 24 days after mTBI. Then, hematoxylin and eosin (H&E) staining was performed as previously described [15]. In brief, tissues were fixed with 10% neutral formalin, embedded in paraffin, and sectioned at 7 μm. Subsequently, sections were deparaffinized with xylene and then stained with hematoxylin and eosin. All sections were evaluated using an AxioVision microscope (Zeiss, Milan, Italy) and the histological results were showed at 20× (50 μm of the Bar scale). All sections were examined for injury with a score from 0 to 3: 0 = no injury, 1 = small focal areas of cell loss, 2 = patchy areas of cell loss in multiple areas of the region, and 3 = tissue infarction.

### 2.7. Copper Staining

Copper staining was performed to determinate copper deposits in tissue sections, according to the manufacturer’s instructions (#KT033, Diagnostic, BioSystems, Pleasanton, CA, USA). In brief, all slides were placed in warmed working rhodanine solution; after two washings in Acetate buffer solution, pH 8.0 for 1 min each, all slides were incubated in hematoxylin for 5 min. After another three washings in acetate buffer solution, slides were cleaned in xylene and then mounted in synthetic resin. All slides were evaluated using light microscopy linked to an imaging system software and images were taken by using objective lens 40× magnification (20 μm of the Bar Scale) (AxioVision and Software; Zeiss, Milan, Italy). The copper deposits were represented in the section by brown granules.

### 2.8. Immunohistochemistry Analysis of NMDAR2B

The immunohistochemical localization was executed as previously described [19]. In brief, all slides were incubated overnight using the following primary antibody: anti-Glutamate receptor (NMDAR2B) (1:100; Thermofisher, Waltham, MA, USA; # MA1-2014). After washing with PBS, sections were incubated with a secondary antibody for 1 h at room temperature. The reaction was revealed by a chromogenic substrate (DAB). The images were acquired using an optical AxioVision microscope (Zeiss, Milan, Italy). For immunohistochemistry, the images were showed at 40× (20 μm of the Bar Scale). 

### 2.9. Enzyme-Linked Immunosorbent Assay (ELISA Kit)

The ELISA kit assay was executed in according to the manufacturer’s protocol on the protein extract of the brain tissues to determine the concentration of: Copper (#ab272528, AbCam, Cambridge, UK), Glutamate transporter-1 (GLT1) (#LS-F6561, LS-Bio, Seattle, USA), GLutamate ASpartate Transporter (GLAST) (#LS-F9806, LS-Bio, Seattle, USA), Zinc transporter 1 (ZnT1) (#LS-F8767, LS-Bio) and Zinc transporter (ZnT3) (#MBS762231, MyBioSource, Kuiper, Netherlands).

### 2.10. Western Blot Analysis of Iba-1, GFAP, CTR1, ATP7A and ATP7B

Western blot analysis was performed on brain tissues harvested 24 days after mTBI, particularly in these brain areas: hippocampus, cortex and other. Cytosolic extract was prepared as described previously [20]. The expression of ionized calcium-binding adapter molecule 1 (Iba-1), glial fibrillary acidic protein (GFAP), copper transporter protein 1 (CTR1), ATPase Copper Transporting Alpha (ATP7A) and ATPase Copper Transporting Beta (ATP7B) was quantified in cytosolic fractions. The filters were probed with specific Abs: anti-Iba-1 (1:500; Santa Cruz Biotechnology, Dallas, TX, USA, SC-32725), anti-GFAP (1:500; Santa Cruz Biotechnology, Dallas, TX, USA, SC-33673), anti-CTR1 (1:500; Santa Cruz Biotechnology, Dallas, TX, USA, SC-66847), anti-ATP7A (1:500; Santa Cruz Biotechnology, Dallas, TX, USA, SC-376467), anti-ATP7B (1:500; Santa Cruz Biotechnology, Dallas, TX, USA, SC-373964) in 1× PBS, 5% *w*/*v* non-fat dried milk, 0.1% Tween-20 at 4 °C, overnight. To ascertain that blots were loaded with equal amounts of proteins, they were also incubated in the presence of the antibody against β-actin protein (cytosolic fraction 1:500; Santa Cruz Biotechnology, Dallas, TX, USA). Signals were detected with enhanced chemiluminescence (ECL) detection system reagent according to the manufacturer’s instructions (Thermofisher, Massachusetts, USA). The relative expression of the protein bands was quantified by densitometry with BIORAD ChemiDocTMXRS + software and standardized to β-actin level. Images of blot signals (8 bit/600 dpi resolution) were imported to analysis software (Image Quant TL, v2003).

### 2.11. Statistical Analysis

All values are expressed as mean ± standard deviation (SD) of n observations. Each analysis was performed three times with three sample replicates for each one. The results were analyzed with GraphPad Prism 9 software by using two-way ANOVA. A *p*-value of less than 0.05 was considered significant (*p* < 0.05).

## 3. Results

### 3.1. PF-06447475 Administration Reduced Brain Tissue Damage after TBI-Induced

To evaluate the tissue damage after TBI, histological examination by hematoxylin and eosin (H&E) staining was performed, particularly observing these brain areas: CA1, dentate gyrus, prefrontal cortex and amygdala [21].

**Figure 1:**
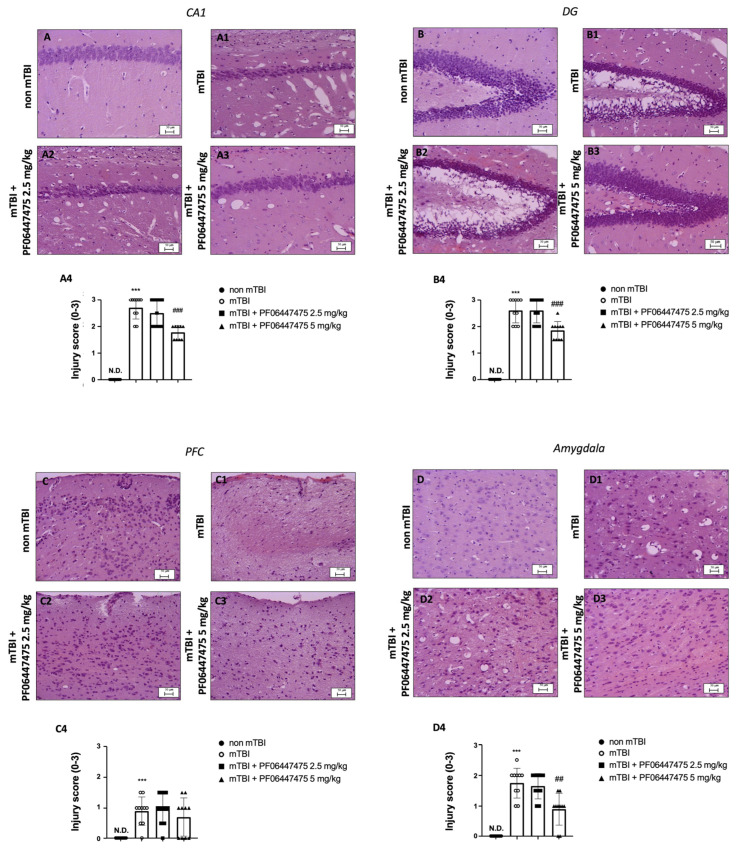
Effects of PF-475 treatments on histological features after mTBI in the CA1, DG, PFC and amygdala areas. CA1 area: H&E staining of the non-mTBI group (**A**) and the mTBI group (**A1**), see injury score (**A4**). PF-475 treatments (2.5 mg/kg (**A2**) and 5 mg/kg (**A3**)), see injury score (**A4**). DG area: H&E staining of the non-mTBI group (**B**) and the mTBI group (**B1**), see injury score (**B4**). PF-475 treatments (2.5 mg/kg (**B2**) and 5 mg/kg (**B3**)), see injury score (**B4**). PFC area: H&E staining of the non-mTBI group (**C**) and the mTBI group (**C1**), see injury score (**C4**). PF-475 treatments (2.5 mg/kg (**C2**) and 5 mg/kg (**C3**)), see injury score (**C4**). Amygdala area: H&E staining of the non-mTBI group (**D**) and the mTBI group (**D1**), see injury score (**D4**). PF-475 treatments (2.5 mg/kg (**D2**) and 5 mg/kg (**D3**)), see injury score (**D4**). Data are expressed as SD from 10 mice for each group. Two-way ANOVA test. (ND) Not detectable. *** *p*< 0.001 vs. non-mTBI; ## *p* < 0.01 and ### *p*< 0.001 vs. mTBI.

In the CA1 area, the first region in the hippocampal circuit, histological analysis revealed relevant damage in the mTBI group, observed through tissue disorganization and white matter alteration associated with restricted zone of small pyramidal cells compared to the non-mTBI group (Figure 1(A1,A), see injury score (A4), *p* < 0.001). Instead, PF-475 treatment at a dose of 2.5 mg/kg, and even more effectively at a dose of 5 mg/kg, significantly decreased the severity of damage (Figure 1(A2,A3), see injury score (A4), *p* 0.36 and 0.007).

**Figure 2:**
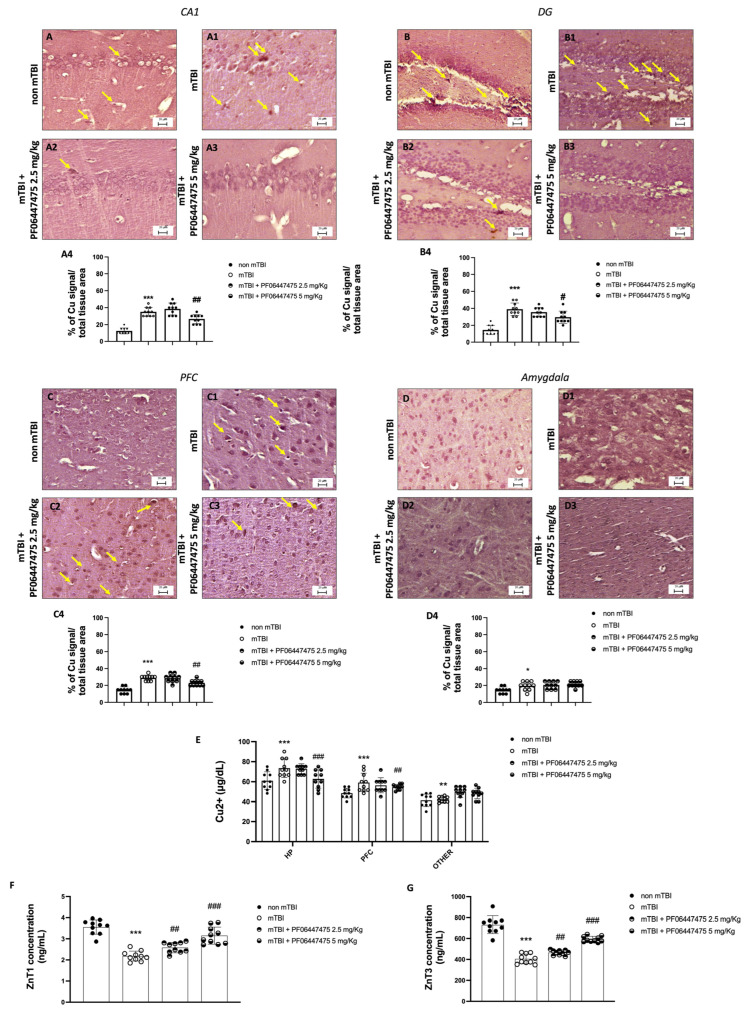
Effects of PF-475 treatments on Cu uptake after mTBI in the CA1, DG, PFC and amygdala areas. CA1 area: copper staining of the non-mTBI group (**A**) and the mTBI group (**A1**), see percentage of Cu signal over the total tissue area (**A4**). PF-475 2.5 mg/kg (**A2**) and 5 mg/kg (**A3**), see percentage of Cu signal over the total tissue area (**A4**). DG area: copper staining of the non-mTBI group (**B**) and the mTBI group (**B1**), see percentage of Cu signal over the total tissue area (**B4**). PF-475 2.5 mg/kg (**B2**) and 5 mg/kg (**B3**), see percentage of Cu signal over the total tissue area (**B4**). PFC area: copper staining of the non-mTBI group (**C**) and the mTBI group (**C1**), see percentage of Cu signal over the total tissue area (**C4**). PF-475 2.5 mg/kg (**C2**) and 5 mg/kg (**C3**), see percentage of Cu signal over the total tissue area (**C4**). Amygdala area: copper staining of the non-mTBI group (**D**) and the mTBI group (**D1**), see percentage of Cu signal over the total tissue area (**D4**). PF-475 2.5 mg/kg (**D2**) and 5 mg/kg (**D3**), see percentage of Cu signal over the total tissue area (**D4**). Copper accumulation (**E**). The concentration of ZnT1 and ZnT3 in brain tissues (**F**,**G**). Data are expressed as SD from 10 mice for each group. Two-way ANOVA test. * *p* < 0.05, ** *p* < 0.01 and *** *p* < 0.001 vs. non mTBI; # *p* < 0.05, ## *p* < 0.01 and ### *p* < 0.001 vs. mTBI.

In the dentate gyrus (DG), a part of the hippocampal formation in the temporal lobe of the brain, histological analysis revealed a significant alteration of tissue morphology, particularly, we observed altered granular cells with dark nuclei, marked disorganization and vacuolization in the mTBI group compared to the non-mTBI group (Figure 2(B1,B), see injury score (B4), *p* < 0.001). PF-475 treatment at the dose of 2.5 mg/kg did not reveal any improvement in injured tissue (Figure 1(B2), see injury score (B4), *p* > 0.99). Instead, PF-475 treatment at the dose of 5 mg/kg significantly decreased the tissue damage, restored granular cells morphology and attenuated vacuolization (Figure 1(B3), see injury score (B4), *p* < 0.001).

In the prefrontal cortex (PFC) area, histological analysis revealed a slight alteration of tissue in the mTBI group compared to the non-mTBI group (Figure 1(C1,C), see injury score (C4), *p* < 0.001). However, PF-475 treatment at the doses of 2.5 and 5 mg/kg did not reveal any improvement in the injured brain (Figure 1(C2,C3), see injury score (C4), *p* 0.82 and *p* 0.43).

In the amygdala area, histological analysis revealed an important damage with white matter alteration and cytoarchitectural alteration in the mTBI group compared to the non-mTBI group (Figure 1(D1,D), see injury score (D4), *p* < 0.001). The treatment with PF-475 at a dose of 2.5 mg/kg did not provide any protection (Figure 1(D2), see injury score (D4), *p* 0.63); on the contrary, PF-475 at a dose of 5 mg/kg decreased the damage and vacuolization (Figure 2(D3), see injury score (D4), *p* 0.001).

The data obtained allow PF-475 treatment at a dose of 5 mg/kg to be considered as a reducer of tissue damage at 24 days after mTBI.

### 3.2. Treatments with PF-06447475 Restored Copper and Zinc Homeostasis following mTBI

Recent studies showed that copper (Cu^2+^) and zinc (Zn^2+^) homeostasis unbalance persists for an extended period following brain injury; in addition, after TBI injury, the Cu^2+^ and Zn^2+^ content gradually increase in the adjacent area to the impact zone, which may contribute to the development of delayed damage in TBI [22]. The molecular mechanisms for the increased Cu uptake in the traumatized brain tissues are related to a nutritional requirement for copper ions for repair of CCI-induced TBI [23]. To evaluate Cu uptake after mTBI, copper staining was performed, particularly observing copper deposits in these brain areas: CA1, dentate gyrus, prefrontal cortex and amygdala.

**Figure 3:**
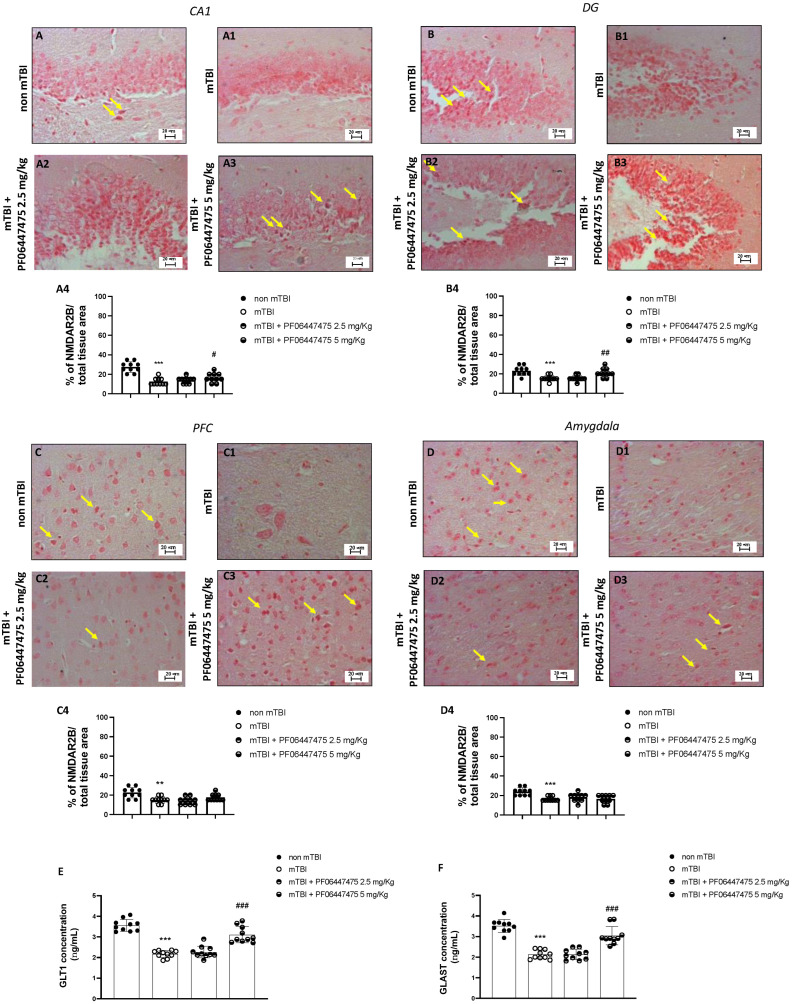
Immunohistochemical localization of NMDAR2B after mTBI in CA1, DG, PFC and amygdala areas. Yellow arrows indicate immunopositively neurons for NMDAR2B antibody. CA1 area: the non-mTBI group (**A**) and the mTBI group (**A1**), see percentage of NMDAR2B over the total tissue area (**A4**). PF-475 2.5 mg/kg (**A2**) and 5 mg/kg (**A3**), see percentage of NMDAR2B over the total tissue area (**A4**). DG area: the non-mTBI group (**B**) and the mTBI group (**B1**), see percentage of NMDAR2B over the total tissue area (**B4**). PF-475 2.5 mg/kg (**B2**) and 5 mg/kg (**B3**), see percentage of NMDAR2B over the total tissue area (**B4**). PFC area: the non-mTBI group (**C**) and the mTBI group (**C1**), see percentage of NMDAR2B over the total tissue area (**C4**). PF-475 2.5 mg/kg (**C2**) and 5 mg/kg (**C3**)**,** see percentage of NMDAR2B over the total tissue area (**C4**). Amygdala area: the non-mTBI group (**D**) and the mTBI group (**D1**), see percentage of NMDAR2B over the total tissue area (**D4**). PF-475 2.5 mg/kg (**D2**) and 5 mg/kg (**D3**), percentage of NMDAR2B over the total tissue area (**D4**). The concentrations of GLT1 and GLAST in brain tissues (**E**,**F**). Data are expressed as SD from 10 mice for each group. Two-way ANOVA test. ** *p* < 0.01 and *** *p* < 0.001 vs. non mTBI; # *p* < 0.05, ## *p* < 0.01 and ### *p* < 0.001 vs. mTBI.

In the CA1 area, copper staining showed an increase of copper deposits in the mTBI group compared to the non-mTBI group (Figure 3(A1,A), see percentage of Cu signal over the total tissue area (A4), *p* < 0.001). PF-475 treatment at a dose of 2.5 mg/kg did not provide any effect (Figure 2(A2), see percentage of Cu signal over the total tissue area (A4), *p* 0.226); on the contrary, PF-475 at a dose of 5 mg/kg significantly decreased Cu uptake, reducing the number of copper deposits (Figure 2(A3), see percentage of Cu signal over the total tissue area (A4), *p* 0.002).

In the DG area, copper staining revealed an important increase of copper deposits in the brain of the mTBI group compared to the non-mTBI group (Figure 2(B1,B), see percentage of Cu signal over the total tissue area B4, *p* < 0.001). Instead, PF-475 treatment at a dose of 2.5 mg/kg, and even more effectively at a dose at 5 mg/kg, significantly decreased copper deposits (Figure 2(B2,B3), see percentage of Cu signal over the total tissue area (B4), *p* 0.24 and *p* 0.09).

In the PFC area, copper staining revealed a slight increase of copper deposits in the mTBI group compared to the non-mTBI group (Figure 2(C1,C), see percentage of Cu signal over the total tissue area (C4), *p* < 0.001). However, PF-475 treatment at the doses of 2.5 and 5 mg/kg did not provide any effect (Figure 2(C2,C3), see percentage of Cu signal over the total tissue area (C4), *p* 0.78 and 0.05).

In the same way, in the amygdala area, staining showed a slight increase of copper deposits in the mTBI group compared to the non-mTBI group (Figure 2(D1,D), see percentage of Cu signal over the total tissue area (D4), *p* 0.02). The treatment with PF-475 at doses of 2.5 and 5 mg/kg did not provide any effect (Figure 2(D2,D3), see percentage of Cu signal over the total tissue area (D4), *p* 0.639 and *p* 0.31).

We also performed an ELISA kit assay to confirm Cu accumulation (Figure 3E).

To check alterations in Zn homeostasis after mTBI, we evaluated the expression of the two most relevant zinc transporters, ZnT1 and ZnT3, which belong to the SLC30A family of Zn^2+^ efflux transporters [24], by ELISA assay. ZnT1 (Figure 2F) and ZnT3 (Figure 2G) levels were noticeably lower in mTBI mice compared to the control group (*p* < 0.001), while the expression levels of zinc transporters both were found to be enhanced after PF-475 treatments at a dose of 2.5 mg/kg (*p* 0.002), and in particular, at the higher dose of 5 mg/kg (*p* < 0.001).

### 3.3. PF-06447475 Treatments Antagonized NMDA Receptor Depletion after mTBI Induction

mTBI-induced cognitive deficits caused by the alteration of receptor molecules are involved in the process of learning and memory [25]. To evaluate the expression of glutamate receptor subunit 24 days after mTBI, immunohistochemistry staining was performed, observing the levels of the extra-synaptic NMDAR2B, particularly in the CA1, dentate gyrus, prefrontal cortex and amygdala brain areas.

In the CA1 area, an important decrease in positive NMDAR2B staining was observed in the mTBI group compared to the non-mTBI group (respectively, Figure 3(A1,A), see percentage of NMDAR2B over the total tissue area (A4), *p* < 0.001). PF-475 treatment at doses of 2.5 and 5 mg/kg did provide a slight effect (Figure 3(A2,A3), see percentage of NMDAR2B over the total tissue area (A4), *p* 0.33 and *p* 0.046).

In the DG area, NMDAR2B positive staining was significantly reduced in the mTBI group compared to the non-mTBI group (respectively, Figure 3(B1,B), see percentage of NMDAR2B over the total tissue area (B4), *p* < 0.001). Instead, PF-475 treatment at a dose of 2.5 mg/kg, and even more effectively at a dose at 5 mg/kg, significantly restored it (Figure 3(B2,B3), see percentage of NMDAR2B over the total tissue area (B4), *p* > 0.99 and *p* 0.005).

In the prefrontal cortex (PFC) area, IHC analysis revealed an important decrease of positive NMDAR2B staining in the mTBI group compared to the non-mTBI group (respectively, Figure 3(C1,C), see percentage of NMDAR2B over the total tissue area (C4), *p* 0.002). However, PF-475 treatment at the doses of 2.5 and 5 mg/kg did not provide any protection (Figure 3(C2,C3), see percentage of NMDAR2B over the total tissue area (C4), *p* 0.55 and *p* 0.12).

At the same, in the amygdala area, NMDAR2B positive staining was significantly reduced in the mTBI group compared to the non-mTBI group (respectively, Figure 3(D1,D), see percentage of NMDAR2B over the total tissue area (D4), *p* < 0.001). The treatment with PF-475 at doses of 2.5 and 5 mg/kg did not provide any protection (Figure 3(D2,D3), see percentage of NMDAR2B over the total tissue area (D4), *p* 0.342 and *p* > 0.99).

Additionally, by checking the expression of the main transporters of glutamate such as GLT1 (Figure 3E) and GLAST (Figure 3F) by ELISA assay, we found a significant decrease of their expression levels in the mTBI mice group when compared to the non mTBI mice group (*p* < 0.001). PF-475 treatment at a dose of 2.5 mg/kg did not provide any significant change (*p* 0.38 and *p* 0.98); otherwise, PF-475 at a dose of 5 mg/kg significantly increased their expression (*p* < 0.001).

### 3.4. PF-06447475 Treatments Attenuated Astrocytes and Microglia Activation and Restored Copper Transporters Expressions (CTR1, ATP7A, and ATP7B)

Activation of microglia and astrocytes contributes to synaptic remodeling, tissue repair and neuronal survival following traumatic brain injury (TBI) [26]. Therefore, we evaluated, by western blot analysis, the expression of Iba-1 and GFAP, as a marker of microglial and astrocyte activation, respectively, in different brain areas (hippocampus, cortex and other).

**Figure 4:**
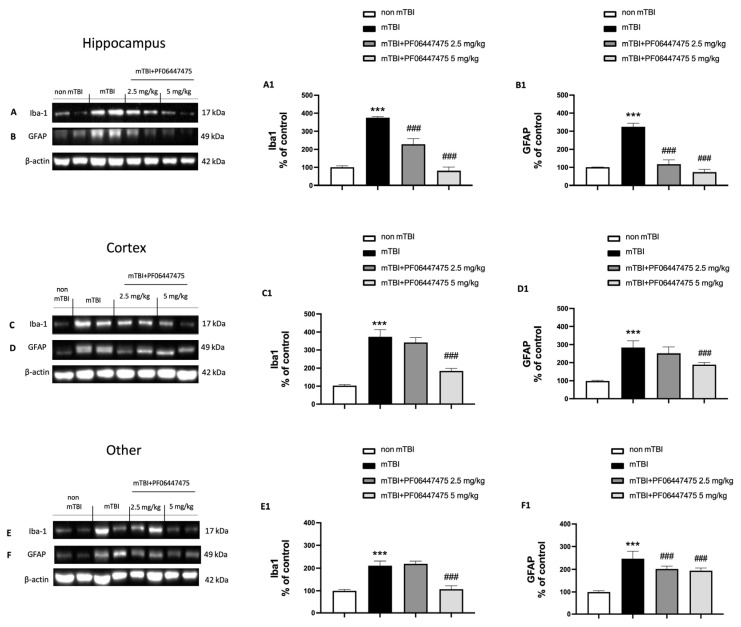
Effects of PF-475 on astrocytes and microglia activation after mTBI in hippocampus, cortex and other. Cytosolic fractions of hippocampus tissue were used to evaluate astrocytes and microglia activation trough the expression of GFAP and Iba-1, respectively. Representative blot of Iba-1 is shown (**A**), see densitometric analysis (**A1**); representative blot of GFAP is shown (**B**), see densitometric analysis (**B1**). Cytosolic fractions of cortex tissue: representative blot of Iba-1 is shown (**C**), see densitometric analysis (**C1**); representative blot of GFAP is shown (**D**), see densitometric analysis (**D1**). Cytosolic fractions of other tissue: representative blot of Iba-1 is shown (**E**), see densitometric analysis (**E1**); representative blot of GFAP is shown (**F**), see densitometric analysis (**F1**). Two-way ANOVA test. *** *p* < 0.001 vs. non mTBI; ### *p* < 0.001 vs. TBI.

In hippocampus we observed a significant increase expression level of Iba-1 and GFAP in the mTBI group compared to the non-mTBI group. The treatment with PF-475 at a dose of 2.5 mg/kg and even more effectively at a dose of 5 mg/kg, significantly reduce their expression (Figure 4 blots A and B, see densitometric analysis A1, *p* < 0.001 and (B1), *p* < 0.001).

Moreover, a significant increase in Iba-1 and GFAP expressions were observed in cortex area from mice subjected to mTBI, while PF-475 treatments reduced their expressions (Figure 4 blots C and D, see densitometric analysis (C1), *p* < 0.001 and (D1), *p* < 0.001).

In other we observed an important increase of Iba-1 and GFAP expressions and treatment with PF-475 was able to decrease their expression to a lesser extent (Figure 4 blots E and F, see densitometric analysis (E1), *p* < 0.001 and (F1), *p* < 0.001).

Additionally, the expression of the main transporters of copper, such as CTR1, ATP7A and ATP7B [27], were evaluated by western blot analysis in the same brain areas (hippocampus, cortex and other).

**Figure 5:**
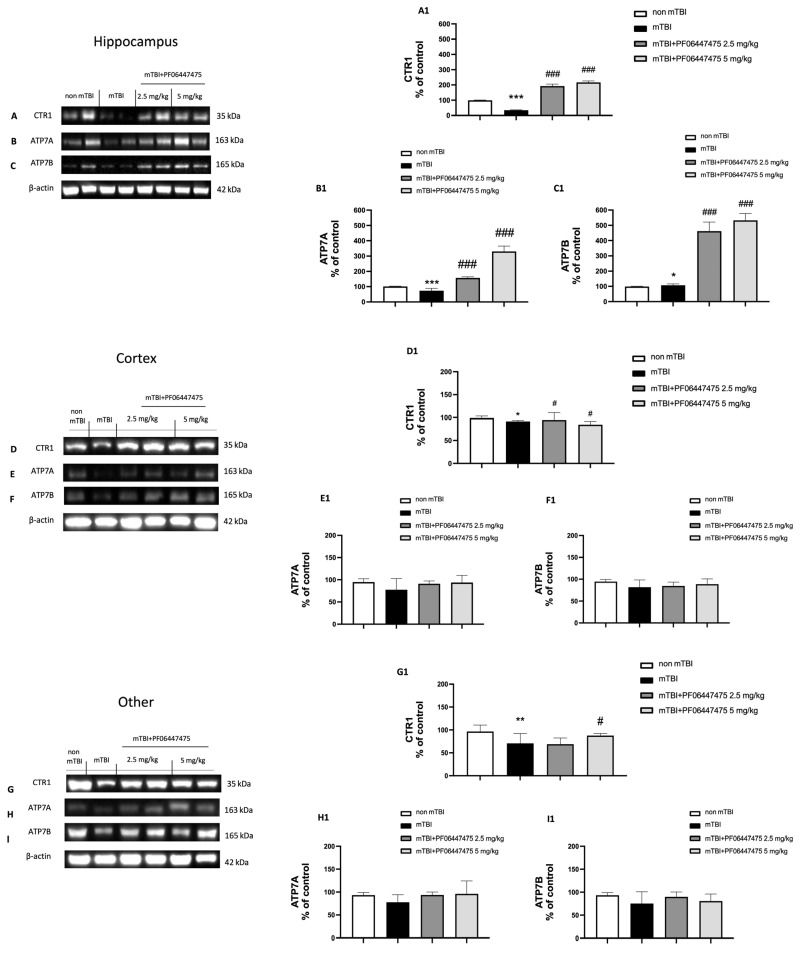
Effects of PF-475 on copper transporters expression after mTBI in hippocampus, cortex and other. Cytosolic fractions of hippocampus tissue were also used to evaluate copper transporters expression, such as CTR1, ATP7A and ATP7B. Representative blots of CTR1, ATP7A and ATP7B are shown (**A**–**C**), see densitometric analysis (**A1**–**C1**). Cytosolic fractions of cortex tissue: representative blots of CTR1, ATP7A and ATP7B are shown (**D**–**F**), see densitometric analysis (**D1**–**F1**). Cytosolic fractions of other tissue: representative blots of CTR1, ATP7A and ATP7B are shown (**G**–**I**), see densitometric analysis (**G1**–**I1**). Two-way ANOVA test. * *p* < 0.05, ** *p* < 0.01 and *** *p* < 0.001 vs. non mTBI; # *p* < 0.05 and ### *p* < 0.001 vs. TBI.

In the hippocampus, we observed that the expression of copper transporters, particularly of CTR1, was significantly reduced after mTBI injury in comparison to the non-mTBI group (*p* < 0.001 and *p* 0.01). PF-475 treatments at doses of 2.5 and 5 mg/kg were able to significantly increase the expression of all copper transporters (Figure 5 blots A–C, see densitometric analysis (A1)–(C1), *p* < 0.001). 

In the cortex, we observed a slight reduction of copper transporters, particularly of CTR1 (*p* 0.01), but in this area, PF-475 treatment did not show an important effect (Figure 5 blots D–F, see densitometric analysis (D1)–(F1), *p* 0.3 and *p* 0.046).

In the same way, in the other, the expression of copper transporters was slightly reduced after mTBI induction (*p* 0.005) and the treatment with PF-475 did not show any significant effect (Figure 5 blots G–I, see densitometric analysis (G1)–(I1)).

### 3.5. Beneficial Effects of PF-06447475 Treatments on Behavioral Consequences of mTBI

The most common assessments of depression-like behaviors in TBI models examine anhedonia, behavioral despair and anxiety [21].

**Figure 6:**
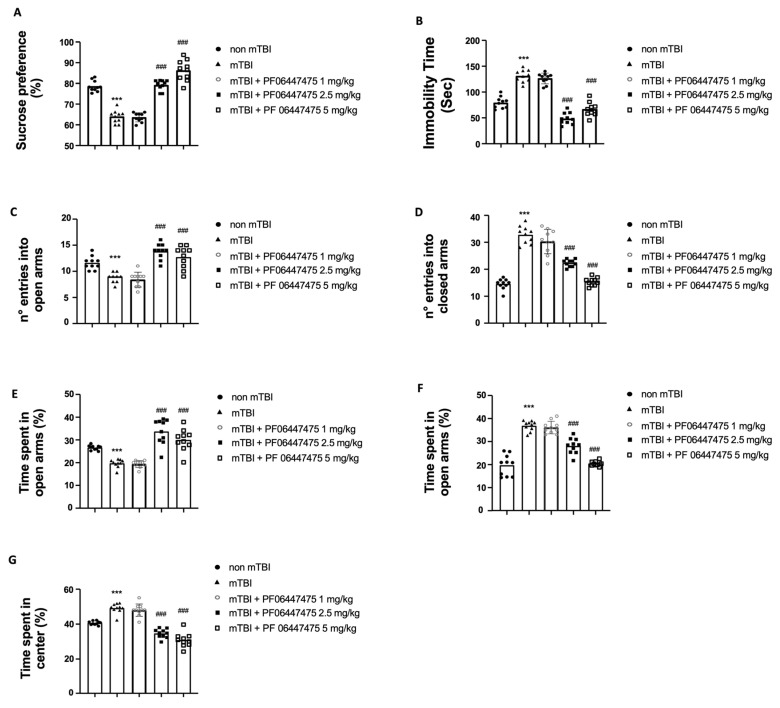
Effect of PF-475 treatment on behavioral function. The sucrose preference test showed that mTBI significantly reduced the percentage of sucrose preference, while PF-475 (2.5 and 5 mg/kg) restored it (**A**). The forced swim test demonstrated that the increased immobility time induced by mTBI in mice was decreased after treatment with PF-475 at doses of 2.5 and 5 mg/kg (**B**). The elevated plus maze test showed that PF-475 treatment (2.5 and 5 mg/kg) reduced depression symptoms after mTBI induction, reducing the number of entries and time spent in closed arms (**D**,**F**); PF-475 increased the number of entries and time spent in open arms (**C**,**E**) and reduced the time spent at center (**G**). Data was expressed as SD from 10 mice for each group. Two-way ANOVA test. *** *p* < 0.001 vs. non mTBI; ### *p*< 0.001 vs. mTBI.

The sucrose preference test (Figure 6A, *p* < 0.001), used to evaluate anhedonia behavior, demonstrated that mTBI induction significantly decreased the percentage of sucrose preference in mice as compared to the non-mTBI group. No significant alteration in the percentage of sucrose preference was found after treatment with PF-475 at a dose of 1 mg/kg in mTBI-induced mice. On the contrary, PF-475 treatments (2.5 and 5 mg/kg) increased the percentage of sucrose preference in mTBI-induced mice, showing anti-depressant-like effects. These findings demonstrate that treatment with PF-475 at doses of 2.5 and 5 mg/kg might alter depression-related symptoms in mTBI-induced mice in the sucrose preference test.

The forced swim test (Figure 6B, *p* < 0.001) was performed to measure behavioral despair. Mice with mTBI displayed a significantly greater immobility time compared to non-mTBI animals in the forced swim test. There were no significant changes in the locomotor activity after PF-475 treatment (1 mg/kg). The results obtained showed that the increased immobility time induced by mTBI in mice was reduced after treatment with PF-475 (2.5 and 5 mg/kg). These data indicate that treatment with PF-475 at doses of 2.5 and 5 mg/kg reduce depression-like behavior in mTBI-induced mice in the forced swim test. 

The elevated plus maze test was used to explore anxiety-like behaviors following TBI. EPM test showed an important increase in the number of entries and the percentage of time spent on closed arms of the mTBI group compared to the non-mTBI group. There were not any significant changes after PF-475 administration at a dose of 1 mg/kg compared with the mTBI group. On the contrary, the treatment with PF-475 (2.5 and 5 mg/kg) showed a reduction in the number of entries and the time spent in closed arms compared to the mTBI group (Figure 6D, *p* < 0.001 and F, *p* < 0.001). 

The EPM test showed a significant decrease in the number of entries and the percentage of time spent in open arms of the mTBI group compared to the non-mTBI group. There were not any significant changes after PF-475 administration at a dose of 1 mg/kg compared with the mTBI group. Instead, the treatment with PF-475 (2.5 and 5 mg/kg) showed an increase in the number of entries and the time spent in open arms compared to the mTBI group (Figure 6C, *p* < 0.001 and E, *p* < 0.001). 

Finally, as shown in Figure 6G (*p* < 0.001), the mTBI group showed an increase in the time spent at center compared to the non-mTBI group. There were not any significant changes after PF-475 treatment at a dose of 1 mg/kg compared with the mTBI group. Instead, the treatment with PF-475 (2.5 and 5 mg/kg) showed a decrease of the time spent at center compared to the mTBI group. These data demonstrated that treatment with PF-475 reduces depression symptoms after mTBI induction. 

Therefore, our data suggests that PF-475 treatment can be used to reduce behavior deficit and depression symptoms after mTBI induction. Based on the behavior test, we decided to only perform the other analyses with PF-475 (2.5 and 5 mg/kg). 

## 4. Discussion

Previously, mTBI was shown to induce profound and long-lasting depression-like symptoms in both humans and mice by inducing neuropsychiatric disorders, even in neurodegenerative diseases such as PD [28]. Recent evidence suggests that mTBI and post-traumatic depression are associated with several anatomic and molecular alterations that include decreased prefrontal grey matter, lesions in the basal ganglia and frontal cortex, increased pro-inflammatory cytokine levels and dysfunction in neurotransmitter systems, including the glutamatergic, dopaminergic and serotonergic systems [29]. Thus far, studies have focused on people with TBI without specifically investigating the association with post-traumatic depression. Therefore, further research is needed to better understand the molecular biomarkers associated with the occurrence of post-traumatic depression after TBI [29]. The mechanisms by which TBI leads to depression are unknown and likely involve multiple brain regions and neurotransmitters [30]. The discovery of the involvement of LRRK2 in PD and its ability to modulate various pro-inflammatory signaling pathways has facilitated hopes of identifying a pharmacological target for neuroprotective therapies that counteract traumatic consequences in the CNS [31]. 

The results of the present study suggest that mTBI induction induces depression-like behavioral changes in mice, as assessed by SPT, FST and EPM [32]. In addition, tissue changes were found in the hippocampus and amygdala, further confirming the depression-like behavior. In the present study, inhibition of LRRK2 by treatment with the antagonist PF-475 showed antidepressant-like activity against mTBI-induced depression-like behaviors and restored the changes in the Cu/NMDAR system.

At day 21 after mTBI, isolated injured mice showed increased depression-like behaviors on scores of the SPT, FST and EPM tests compared to mice without mTBI. To confirm depression-like symptoms after mTBI, SPT was used to test for anhedonia. Anhedonia is the condition in which pleasure is not felt when ingesting sucrose. Our results showed that mTBI significantly reduced sucrose intake and promoted anhedonia in mice. Although mice treated with PF-475 1 mg/kg did not show a significant increase in sucrose preference after mTBI and were defined as non-anhedonia, the reduction in sucrose intake in mice exposed to mTBI was reversed by PF-475 treatments in a significantly dose-dependent manner. Indeed, higher doses of 2.5 and 5 mg/kg increased the pleasure of drinking sweet water by counteracting anhedonia and depressive symptoms. Behavioral analysis of the groups of mice revealed that anhedonia in mTBI mice is associated with other features of depressive-like behavior, such as increased hovering in the FST and increased anxiety in the EPM, the most commonly used behavioral tests to study the effects of antidepressants, which parallel the development of anhedonia [33,34]. In the FST, we observed that mTBI mice showed a significant increase in immobility time, suggesting that depression-like behaviors were caused by long-term consequences of traumatic brain exposure. Indeed, our results showed that treatment with PF-475 had an antidepressant effect in the FST by decreasing the immobility time of the mice. As for the anxiety-like behaviors, at 21 days after mTBI, the mice did not prefer open arms, but preferred to stay in closed arms, where they felt safer and more isolated, releasing their emotional and physiological manifestations of depression, while inhibition of LRRK2 kinase activity by 14 days of treatment with the antagonist PF-475 facilitates exploration of the open arm of the EPM test and alters the tendency in the mice to react rather than remain at rest. These observations are confirmed by numerous clinical studies indicating that the reduction of LRRK2 activity by small molecule antagonists is neuroprotective.

From a neuropathological perspective, in addition to the macroscopic and significant lesion in the cortex of mTBI mice, developing neuronal damage was observed in other depression-related brain regions such as the hippocampus and amygdala [35]. In this study, we observed relevant damage in the mTBI group, as evidenced by tissue disorganization and white matter alteration in the CA1 and amygdala areas. Histological evaluation showed that treatment with PF-475 led to a reduction in lesion area and severity of damage.

Scientific evidence supports the involvement of zinc and copper in the development of neurodegenerative diseases. Zinc is involved in many central nervous system (CNS) diseases, such as traumatic brain injury, ischaemia and mood disorders, including depression [36]. Under pathological conditions, including mTBI, the excess zinc is rapidly released from the presynaptic neuronal vesicles, crosses the postsynaptic membrane and causes neuronal damage and death [37]. Indeed, zinc transporters are therefore critical for maintaining ion homeostasis and, in agreement with this evidence, our results demonstrated a lower expression of zinc transporters (ZnT1 and ZnT3) following mTBI. In CNS, copper is involved in the myelination process and is able to modulate synaptic activity and excitotoxic cell death [38]. Studies conducted on the mTBI model have shown that the Cu^2+^ content gradually increases in the area adjacent to the impact zone, contributing to the development of delayed damage in the TBI [9]. Copper deposits, in particular in the CA1 and DG areas, restore copper homeostasis. Zinc transporters are therefore crucial for the maintenance of ion homeostasis, and in line with this finding, our results showed lower expression of zinc transporters (ZnT1 and ZnT3) after mTBI. In the CNS, copper is involved in the myelination process and may modulate synaptic activity and excitotoxic cell death [38]. Studies using the mTBI model have shown that Cu^2+^ content gradually increases in the area adjacent to the impact zone, contributing to the development of late damage in TBI [9]. Indeed, we found that mTBI was characterized by an increase in copper deposition, but PF-475 treatments significantly reduced copper deposition, especially in the CA1 and DG areas, restoring copper homeostasis. Copper transporters (ATPases) are essential for the regulation and maintenance of copper-mediated processes in the brain; their dysfunction leads to severe neurological deficits and neurodegeneration. Consequently, functional defects in copper ATPases may contribute to the development of neurodegenerative diseases in which copper is dysregulated, such as Alzheimer’s disease [39]. Accordingly, our data showed that after mTBI, the expression of copper transporters (CTR1, ATP7A and ATP7B) strongly decreased. In contrast, treatment with PF-475 increased their expression levels, especially in the hippocampus, confirming the ability of PF-475 to restore copper homeostasis [27]. Copper also interacts with glutamatergic and GABA-ergic synapses; stimulation of the NMDA receptor (NMDA-R) induces the release of copper in hippocampal neurons and is associated with repositioning of the ATP7A transporter to hyperactive sites [40]. Recent studies suggest that TBI and MDD are characterized by dysregulation of glutamate homeostasis [30]. Thompson et al. observed a decrease in markers of AMPA activity in some regions, including the prefrontal cortex and hippocampus, following chronic stress [41]. Similarly, the results of our study show a lower expression of NMDAR2B, a glutamate receptor, after mTBI. In addition, our data showed a significant decrease in the expression of glutamate transporters (GLT1 and GLAST); similarly, Piao et al. indicated a decrease in glutamate transporters after mTBI [30]. Therefore, these findings may indicate that glutamatergic disturbances after TBI increase susceptibility to depression (MDD) [42].

In the CNS, microglia and astrocytes are involved in supporting functions, including energy metabolism, synaptic plasticity and ion homeostasis. Under pathological conditions such as traumatic brain injury, a neuroinflammatory state occurs that causes glial activation. In particular, the increase in reactive astrocytes and microglia leads to a high expression of GFAP and Iba-1, respectively. Therefore, GFAP and Iba-1 are also reliable biomarkers for brain damage in the context of neurodegenerative diseases. Our results clearly confirmed a strong change in GFAP and Iba-1 expression after TBI-induced degeneration. However, treatments with PF-475 were able to significantly decrease their expression, reduce reactive astrocytes and microglia, and regulate GFAP and Iba-1 levels. Similarly, Piao et al. confirmed our findings in their study of an increase in GFAP expression in the hippocampus seven days after TBI [30].

The results obtained indicated that mTBI induction enhanced the production of depression-like behavioral changes, which was confirmed by tissue changes in the hippocampus and amygdala. Inhibition of LRRK2 by PF-475 showed an antidepressant effect against mTBI-induced depressive behaviors and restored copper homeostasis and NMDAR expression.

## 5. Conclusions

In conclusion, this study suggests that LRRK2 may represent a new therapeutic strategy for patients with depression-related symptoms. This is because mTBI leads to persistent cognitive impairments that begin weeks to months after injury. The deficits in emotionality and learning plasticity of the injured mice, localized in different brain regions such as the hippocampus, amygdala and prefrontal cortex, suggest that targeting LRRK2 could improve both behavioral and learning and memory impairments in clinical populations with moderate traumatic brain injury. A better understanding of the activity of LRRK2 is necessary to determine the therapeutic use of its inhibitors, particularly for the long-term treatment of CNS disorders.

## Data Availability

The data that support the findings of this study are available from the corresponding author upon reasonable request.
