# Correction: Filippone et al. Inhibition of LRRK2 Attenuates Depression-Related Symptoms in Mice with Moderate Traumatic Brain Injury. *Cells* 2023, *12*, 1040

**DOI:** 10.3390/cells15111017

**Published:** 2026-06-01

**Authors:** Alessia Filippone, Laura Cucinotta, Valentina Bova, Marika Lanza, Giovanna Casili, Irene Paterniti, Michela Campolo, Salvatore Cuzzocrea, Emanuela Esposito

**Affiliations:** Department of Chemical, Biological, Pharmaceutical and Environmental Sciences, Viale Stagno d’Alcontres, 31, 98166 Messina, Italy; afilippone@unime.it (A.F.); laura.cucinotta@unime.it (L.C.); valentina.bova@unime.it (V.B.); mlanza@unime.it (M.L.); gcasili@unime.it (G.C.); ipaterniti@unime.it (I.P.); campolom@unime.it (M.C.); salvator@unime.it (S.C.)

## Error in Figure

In the original publication [1], there was a mistake in Figure 3 as published. The mistake identified in Figure 3 (panels B3 and D2, respectively) is attributable to an unintentional error that occurred during the figure assembly. From the original raw data and source files, we confirm that the error was exclusively restricted to the assembly/presentation of the specific panel in Figure 3. The underlying experimental data used for quantification, normalization, statistical analysis, and graphical representation were generated independent of the mistakenly inserted image and were derived from the correct original datasets. Also, the quantification reported in Figure 3 was performed using the correct raw data; the statistical analyses associated with Figure 3 remain unchanged (1); no other figures, supplementary figures, or conclusions of the manuscript are affected by this error.

Therefore, the error does not impact the validity of the quantitative results, the interpretation of the experiment, or the overall conclusions of the study.

To ensure full transparency, we have corrected Figure 3. We have also carefully re-checked all figures in the manuscript to exclude the presence of additional assembly errors.

The authors state that the scientific conclusions are unaffected. This correction was approved by the Academic Editor. The original publication has also been updated.

The revised Figure 3 appears below.

## Figures and Tables

**Figure 3:**
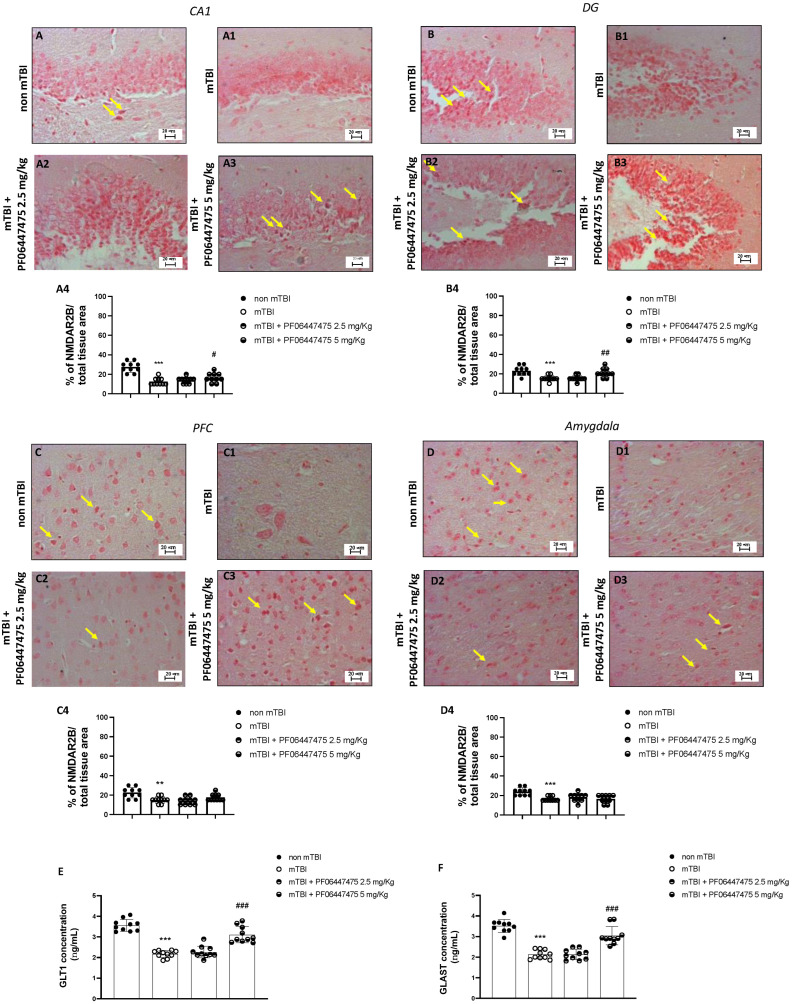
Immunohistochemical localization of NMDAR2B after mTBI in CA1, DG, PFC and amygdala areas. Yellow arrows indicate immunopositively neurons for NMDAR2B antibody. CA1 area: the non-mTBI group (**A**) and the mTBI group (**A1**), see percentage of NMDAR2B over the total tissue area (**A4**). PF-475 2.5 mg/kg (**A2**) and 5 mg/kg (**A3**), see percentage of NMDAR2B over the total tissue area (**A4**). DG area: the non-mTBI group (**B**) and the mTBI group (**B1**), see percentage of NMDAR2B over the total tissue area (**B4**). PF-475 2.5 mg/kg (**B2**) and 5 mg/kg (**B3**), see percentage of NMDAR2B over the total tissue area (**B4**). PFC area: the non-mTBI group (**C**) and the mTBI group (**C1**), see percentage of NMDAR2B over the total tissue area (**C4**). PF-475 2.5 mg/kg (**C2**) and 5 mg/kg (**C3**)**,** see percentage of NMDAR2B over the total tissue area (**C4**). Amygdala area: the non-mTBI group (**D**) and the mTBI group (**D1**), see percentage of NMDAR2B over the total tissue area (**D4**). PF-475 2.5 mg/kg (**D2**) and 5 mg/kg (**D3**), percentage of NMDAR2B over the total tissue area (**D4**). The concentrations of GLT1 and GLAST in brain tissues (**E**,**F**). Data are expressed as SD from 10 mice for each group. Two-way ANOVA test. ** *p* < 0.01 and *** *p* < 0.001 vs. non mTBI; # *p* < 0.05, ## *p* < 0.01 and ### *p* < 0.001 vs. mTBI.
